# Predicting Treatment Outcomes Using Explainable Machine Learning in Children with Asthma

**DOI:** 10.3390/children8050376

**Published:** 2021-05-10

**Authors:** Mario Lovrić, Ivana Banić, Emanuel Lacić, Kristina Pavlović, Roman Kern, Mirjana Turkalj

**Affiliations:** 1 Knowledge Discovery, Know-Center, Infeldgasse 13, 8010 Graz, Austria; mlovric@know-center.at (M.L.); elacic@know-center.at (E.L.); kpavlovic@know-center.at (K.P.); 2 Srebrnjak Children’s Hospital, Srebrnjak 100, 10000 Zagreb, Croatia; ibanic@bolnica-srebrnjak.hr (I.B.); mturkalj@bolnica-srebrnjak.hr (M.T.); 3 Institute of Interactive Systems and Data Science, Graz University of Technology, Inffeldgasse 16C, 8010 Graz, Austria; 4 Faculty of Medicine, J.J. Strossmayer University of Osijek, Josipa Huttlera 4, 31000 Osijek, Croatia; 5 Medical School, Catholic University of Croatia, Ilica 242, 10000 Zagreb, Croatia

**Keywords:** asthma control, asthma controller medication, childhood asthma, machine learning, treatment outcome

## Abstract

Asthma in children is a heterogeneous disease manifested by various phenotypes and endotypes. The level of disease control, as well as the effectiveness of anti-inflammatory treatment, is variable and inadequate in a significant portion of patients. By applying machine learning algorithms, we aimed to predict the treatment success in a pediatric asthma cohort and to identify the key variables for understanding the underlying mechanisms. We predicted the treatment outcomes in children with mild to severe asthma (*N* = 365), according to changes in asthma control, lung function (FEV1 and MEF50) and FENO values after 6 months of controller medication use, using Random Forest and AdaBoost classifiers. The highest prediction power is achieved for control- and, to a lower extent, for FENO-related treatment outcomes, especially in younger children. The most predictive variables for asthma control are related to asthma severity and the total IgE, which were also predictive for FENO-based outcomes. MEF50-related treatment outcomes were better predicted than the FEV1-based response, and one of the best predictive variables for this response was hsCRP, emphasizing the involvement of the distal airways in childhood asthma. Our results suggest that asthma control- and FENO-based outcomes can be more accurately predicted using machine learning than the outcomes according to FEV1 and MEF50. This supports the symptom control-based asthma management approach and its complementary FENO-guided tool in children. T2-high asthma seemed to respond best to the anti-inflammatory treatment. The results of this study in predicting the treatment success will help to enable treatment optimization and to implement the concept of precision medicine in pediatric asthma treatment.

## 1. Introduction

The aim of personalized medicine is to provide a target therapy for each individual or phenotype, based on the corresponding syndrome or disease [1]. Even though machine learning techniques have identified a number of structures and/or phenotypes in asthma, one has to be careful in the clinical interpretation of these structures, as they may not represent true endotypes (underlying immunopathological mechanisms) [2]. Overlaps in the endotypes, as well as the clinical presentation of the disease, make the delivery of a personalized asthma treatment quite elusive [3,4]. Furthermore, the same pattern of symptoms does not necessarily indicate the same underlying mechanism, and moreover, different mechanisms are not mutually exclusive and may even act synergistically. The emergence of machine learning algorithms, the abundance of clinically significant data and computing power can be attributed as key enablers in the development of personalized medicine. There is a substantial body of scientific work on data-driven methods in asthma phenotyping, and the variables, as well as the model chosen, can largely affect the models and obtained results [5,6,7,8,9,10,11,12,13]. A careful selection of both the predictive model and the dataset are essential in such studies, with expert clinical interpretation being of the utmost importance [14]. In childhood asthma, inhaled corticosteroids (ICS) (in combination with long-acting beta-agonists, LABA and/or add-on leukotriene receptor antagonists, LTRA) remain the controller medications of choice, although evidence shows that a significant proportion of patients fail to respond adequately to such treatment [5,6,7,8]. The complexity of the disease or, better said, the “umbrella” diagnosis of asthma that encompasses a number of different phenotypes underpinned by different pathophysiological mechanisms or distinct inflammatory pathways (endotypes) seems to be the major obstacle in asthma management, as well as in the development of personalized treatment approaches [9]. An important study on prediction was conducted by Belgrave et al. [10], focusing on preschool wheezers (*N* = 150) with a large dataset (*N* (variables) = 636) using selected state-of-the-art techniques for data processing and machine learning and obtained 90%+ performances in Kappa statistics. The authors also reported the robustness and performance quality when using Random Forest and that subjective variables are important in distinguishing ill patients from controls. Another important study focusing on the asthma control-based response to controller medication was conducted in the Childhood Asthma Management Program (CAMP) cohort using novel machine learning algorithms [11]. They reported that asthma control, a bronchodilator response and serum eosinophils were the most predictive variables in asthma control, regardless of the medication used. Luo et al. [12] demonstrated that machine learning studies in asthma rarely deal with predictive models in clinical practice.

### Our Aim and Contribution

The primary aim of our research was to test the predictive possibilities for treatment success after 6 months of medication use in pediatric asthma patients and reveal the key variables for understanding the mechanisms underlying such responses using machine learning algorithms. Identifying non-responders vs. responders to a treatment with machine learning tools such as those employed in this study is essential in asthma management, as predicting treatment failure is extremely hard (if not impossible) in the current practice. Our study was based on an observational childhood asthma cohort that was ethnically homogeneous, age diverse and reflected real-life clinical situations, with the majority of patients having mild-to-moderate disease. The main outcomes were four different parameters of the response to treatment (after 6 months) assessed by changes in the lung functions and level of control. Each of these targets was evaluated at baseline (t0) and after 6 months (t0 + 6) of treatment use, alongside the other parameters and biomarkers. The predictive possibilities were tested by means of machine learning, i.e., Random Forest and AdaBoost classification algorithms, where the treatment outcomes (as binary variables) were set as values to be predicted based on other clinically relevant data and assessments. Since such algorithms are considered black box models, understanding ML models is of the utmost importance in medicine [15]. For this reason, we introduced model explainability by the use of permutation importance for understanding the most important variables for differentiating between responders and non-responders.

## 2. Materials and Methods

### 2.1. Population Studied

Three hundred and sixty-five pediatric patients (355 children aged 2–17 and ten adolescents aged 18–22) with atopic and nonatopic, mild to severe persistent asthma [16] were recruited in a prospective, noninterventional type of clinical study at the Srebrnjak Children’s Hospital outpatient clinic (Zagreb, Croatia). Informed consent was obtained from the children’s parents/legal guardians. The study protocol was approved by the local Ethics Committee (No. 6/2015, 5 June 2015). All patients underwent a physical examination, anthropometric measurements and standard diagnostic procedures to establish a diagnosis of asthma and guide its management (Table 1). The patients started treatment with ICS (alone or in combination with LABA) and/or LTRA, according to the disease severity and previously assessed level of disease control. A follow-up visit with lung function and airway inflammation testing was made after 6 months of treatment use. Additionally, treatment outcomes and the level of asthma control (according to the Global Initiative for Asthma, GINA [16]) were assessed at the follow-up visit. In total, 280 variables (observations) were collected. This observational study is described in the supplementary file in detail.

### 2.2. Response Variables

For a machine learning experiment, one needs predictive (X matrix) and response or target variables (y). In this work, we used four target variables that represent the treatment responses (outcomes) to asthma medication, namely (i) forced expiratory volume in one second (FEV1) and (ii) maximal expiratory flow at 50% of the vital flow capacity (MEF50), (iii) changes in airway inflammation (Fractional Exhaled nitric oxide (FENO)) and (iv) the level of asthma control (LOAC) assessed by a pediatric pulmonologist. According to their response to treatment after 6 months of medication use, the patients were divided into “responders” and “non-responders” in accordance with the Minimal Clinically Important Difference (MCID) for lung function adjusted for children (% of the predicted FEV1 and MEF0) and data from other studies, taking into account changes in the level of asthma control (LOAC) and changes in the FENO [17,18,19,20,21]. The response variables are described in Table 2.

### 2.3. Data Preparation and Balancing

We used Python scripts and the methods previously described for data processing and modeling [22]. Predictive variables with more than 10% missing values were removed. Variables with fewer than 10% missing values were imputed by their respective median for continuous variables or mode for discrete variables. To avoid the “curse of dimensionality”, where models suffer from an overly large number of predictive variables [23], we aggregated the individual variables describing allergic sensitization (skin prick test (SPT) and allergen-specific immunoglobulin E (sIgE) test results). These variables were binarized and summed into four categories: seasonal inhaled, perennial inhaled, insect venom and food allergens. Strong sensitization to house dust mite, cat dander and ragweed were treated separately due to their association with disease severity and more severe outcomes [24,25]. The dataset for predicting treatment outcomes consisted of 365 patients and 73 patient-related variables (listed in the supplementary file) and four target variables (LOAC, FEV1, FENO and MEF50). We dealt with an imbalanced classification problem (see Table 3), i.e., responders (1) or non-responders (0) could have been underrepresented. Imbalanced classification models tend to recognize the major class better while struggling with the often-scarce minor class, meaning that predictions may be biased towards the major class [22,26]. To avoid this, we employed synthetic data generation techniques, namely oversampling (OS) and under sampling (on the training set exclusively). Oversampling was conducted by means of SMOTE [27], which was previously reported for improving the performance on predicting lung disease outcomes [10]. We utilized Cluster Centroids (CC) [28,29] for under sampling.

### 2.4. Machine Learning

**Figure 1:**
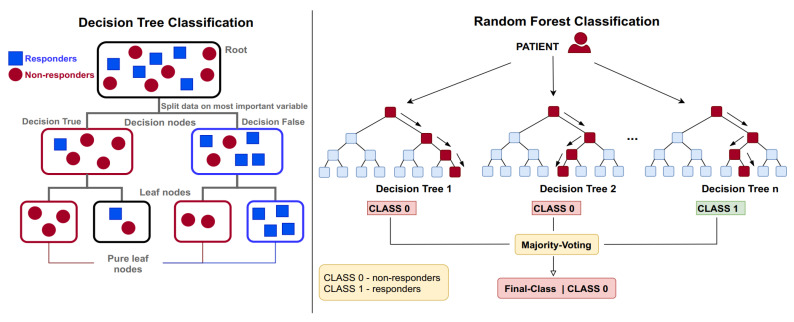
(**Left**) A typical (weak) tree classifier. A group of patients with both responders and non-responders is to be separated based on the given predictive variables. The first split happens with a variable that gives the best split for the two groups. The algorithms split the groups until it gets leaf nodes (tree bottom) as pure as possible for the aimed classes. (**Right**) Simplified scheme of the random forest algorithm. The trees represent weak classifiers that are aggregated via voting to form a strong one. Every tree trains on a random part of the training data (bootstrapping). The AdaBoost classifier trains the trees sequentially instead of parallel.

Our aim was to estimate to which class a patient belongs (responder or non-responder) after 6 months of treatment (t0) based on 73 predictive variables for 365 patients. The employed ensemble classification algorithms follow a paradigm where multiple “weak nonlinear classifiers” are trained and averaged to improve the prediction abilities and lower the prediction error (Figure 1). In problem settings such as the present one, nonlinear classifiers are the solution of choice, since linear classifiers tend to fail with heterogeneous data [30]. Furthermore, the use of tree-based classifiers reduces the workload with data preprocessing and utilizes less variables, since the categorical ones do not need to be binary-encoded. A review of the advantages in using tree classifiers in medicine is given in Reference [30].

We used two types of machine learning classification algorithms: the AdaBoost (AB) [31] nd Random Forest [32] (RF) classifiers. The data was split [26] into train (75%) and test (validation) sets (25%). Due to the imbalance, the minor class was stratified respectively per target to have an equal distribution of the minor class in training and the test set. The experimental matrix for the machine learning models is described in Table 4. For optimizing the models in a large parameter space, we also used 5× cross-validation in the train set; however, to show the true performances of the classifiers, we reported model metrics on the test sets.

For model explanation and variable selection, we used permutation importance (PI) [33], which we used in our prior work [34]. Permutation importance refits pretrained models in each run and permutes (or shuffles) the values of the individual predictive variables. If a variable was important to the model, the model quality will deteriorate if the values in the variable are shuffled. A drop in the model quality is given as a weight towards the quality metrics. The higher the dependence of the model on a variable, the higher its weight. Variable selection is utilized by selection features that are above 0 permutation importance. The model quality metrics used in this work were Accuracy, Sensitivity, Specificity and the Matthews correlations coefficient (MCC) [35,36]. A detailed description of these metrics is given in the supplementary data (Table S4).

## 3. Results

### 3.1. Performance in Prediction of Treatment Outcomes

Table 5 presents the best achieved classification results for each particular treatment outcome. Overall, the MCC metrics, which are sensitive to imbalanced classification and random effects, show the classification results are above random. The highest prediction accuracy is achieved for LOAC. This is due to a high number of correctly predicted outcomes for responders (indicated by specificity) and non-responders (indicated by sensitivity), as well as a high MCC.

When predicting a response according to FEV1 (t0 + 6), FENO (t0 + 6) and MEF50 (t0 + 6), an average accuracy between 65% and 70% was achieved. Compared to lung function-based treatment outcomes (MEF50 and FEV1), predicting the outcomes assessed by changes in FENO (baseline to 6m) performed better, as it showed a slightly higher accuracy and much better sensitivity (good prediction performance for responders). However, for treatment outcomes according to lung function and FENO, a much lower MCC (21–26%) was achieved when compared to LOAC (t0 + 6).

**Figure 2:**
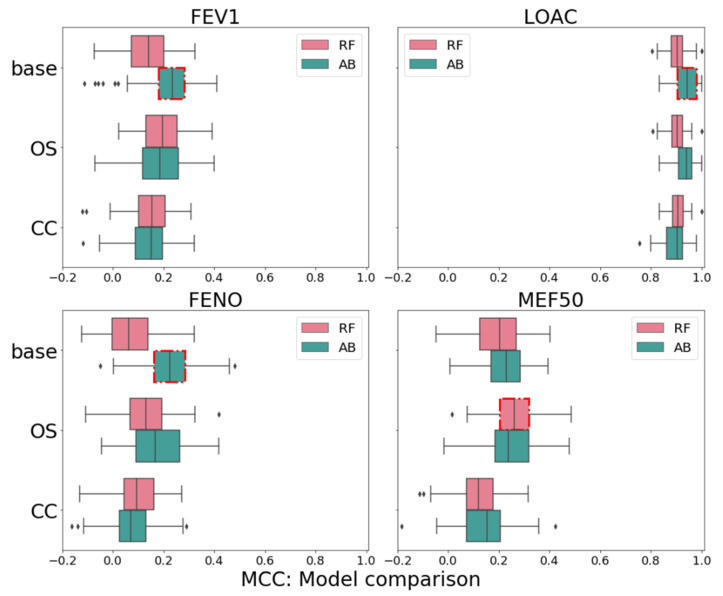
Boxplot of the classification results by means of the MCC (x-axis). The comparison includes the classification results for the four targets (FEV1, LOAC, FENO and MEF50); two classification algorithms (AB—Ada Boost, RF—Random Forest) and two sampling methods (Oversampling: OS and Cluster Centroids: CC) compared to no sampling (base). The best models are assigned per target by a red square surrounding the box. FEV1—forced expiratory volume in one second, MEF50—maximal expiratory flow at 50% of the vital flow capacity, FENO—Fractional Exhaled nitric oxide, LOAC—level of asthma control, MCC—Matthews correlation coefficient.

We utilized two different classification methods and three sampling techniques. Figure 2 shows the distribution of the model results by means of the MCC across the sampling methods and classifiers. AdaBoost was the better-performing classifier for LOAC, FEV1 and FENO, except for MEF50 (t0 + 6), where RF outperformed it marginally. This is of no surprise, since boosting algorithms generally show good performances with imbalanced sets [37]. Overall, not sampling the data, in our case, led to the best prediction results in combination with the AdaBoost algorithm. Using oversampling resulted in a better MCC only for MEF50 (t0 + 6), which could indicate that, when designing experiments like these, one has to take care of nonuniform variable spaces for the rare or minor classes like the responders here [37]. Even though the differences are marginal, in medicine, even the slightest improvement may be important. These results can be explained by the advantage of AdaBoost, which learns sequentially on the misclassification from previous weak learners in the sequence, and while over/under sampling improves the results for RF, it is not the case with AdaBoost. Additionally, since RF is trained in parallel, it is much faster in practice; hence, for training fast and large datasets, RF with oversampling could be used.

### 3.2. Model Interpretation

For each target modeled in the experimental matrix, we calculated the average permutation importance (PI) for the predictive variables (Table 6). The only predictive variable passing the 1% threshold for the classification of LOAC outcomes is “asthma severity”.

**Figure 3:**
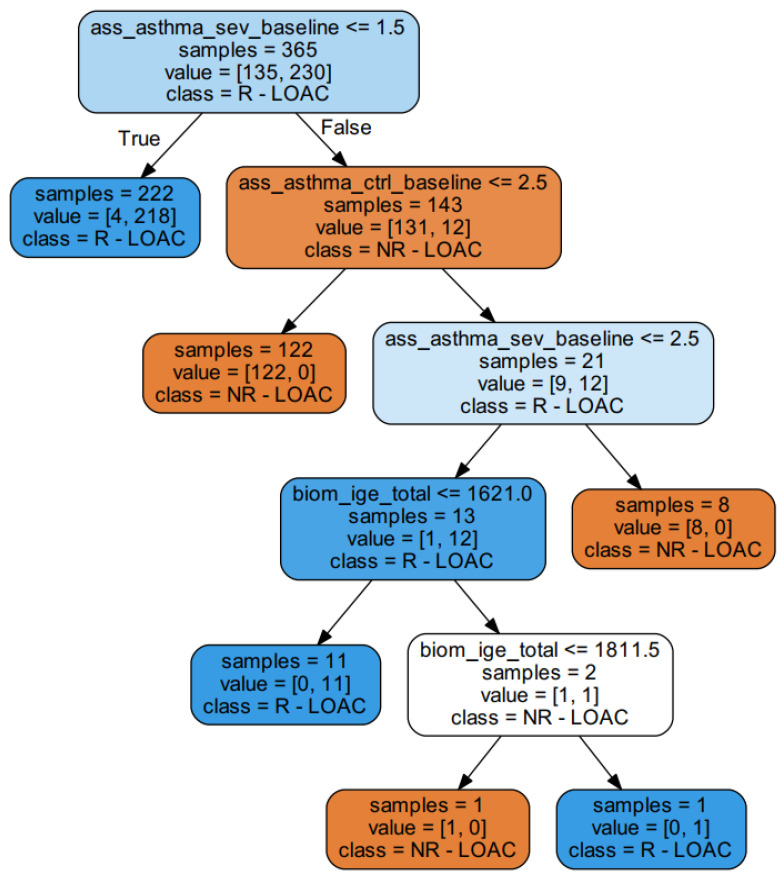
An exemplary decision tree classifier where the treatment outcome LOAC after six months was predicted by three predictive variables (LOAC baseline, Asthma severity baseline and IGE_total). The responders are assigned as R-LOAC (responders by level of asthma control), while the non-responders are assigned as NR-LOAC (non-responders by level of asthma control). The asthma severity baseline is the first split. Most of the responders will respond well to treatment if their asthma severity was estimated to have a value of 1. In an ensemble classifier, a few hundred of these are trained on the bootstrapped samples and averaged for prediction, which is explained in Figure 1. Ass_asthma_sev_basline: asthma severity (according to GINA) grade assessed at baseline, ass_asthma_ctrl_basline: asthma control assessed at baseline and biom_ige_total: total serum IgE.

Additionally, LOAC appears to be associated with the least complexity in regard to prediction, with only one important predictor, in spite of its great power of prediction (Table 6). To understand this, we submitted this subset and the target LOAC to a decision tree classifier (Figure 1, left) to follow the decisions of complex classifiers that consist of many such weak classifiers; see Figure 3. This exemplary decision tree (only a subpart of an ensemble classifier) shows that children with milder forms of the disease respond well to treatment with ICS, as well as that severe patients do not respond to treatment adequately, i.e., severe patients at the baseline remain uncontrolled after 6 months of medication use.

To understand the age effects, known to be an important factor in disease pathophysiology, we stratified the predicted data in the test sets into age groups (2–5, 6–11, 12–17 and 18+) and calculated the average MCC per group to identify whether age might influence the predictive quality of the models. The results are given in Table 7. The best results for LOAC-based outcomes were achieved for young children (2–5 years), with a perfect MCC of 1.0, while, for the adolescent group, the approach failed, with an MCC of 0.29. Similar observations are presented for MEF50 with the 2–5-year-old age group having the best and 18+ the worst results (MCC 0.29 vs. 0). The group of 6–11 years of age showed the best results for FENO- (MCC 0.14) and FEV1 (MCC 0.27)-based outcomes.

### 3.3. The Need for Machine Learning

There is an ongoing discussion whether to use black box models in modeling scenarios [38,39,40,41], such as the used cases presented here as black box models, and their decisions cannot be easily understood. For this reason, we compared the model results of the ensemblers to logistic regression, which was treated in the same manner as the ensemble models, i.e., split 100 times randomly and optimized during cross-validation in the train set. Logistic regression can be considered a baseline due to its simplicity and interpretability (explainability) coming from simple splits in data space [42] and is often used in asthma studies [13,43,44,45]. The comparison is given on the test set as the median values in Table 8.

## 4. Discussion

To justify the use of machine learning algorithms, we compared our models to logistic regression. Our comparison shows that an algorithm relying on a linear combination of variables fails on average against ensemble algorithms. The low performance of the linear model was also observed in our prior and other works regarding both regression [41,46] and classification tasks [30,44,47]. Besides failing due to nonlinear relationships and boundaries between the classes, linear models can also suffer from the utilization of irrelevant features in models and complex cancellation effects [30].

Although LOAC is a less objective variable than lung function and FENO, as it encompasses symptom self-assessment, it reflects real-life treatment success best. This is in concordance with the control-based management approach, focusing on achieving adequate control of the symptoms and minimizing the future risks of exacerbations [16]. The lower predictive quality with FEV1 and MEF50 suggests that lung function is not a preferred tool to be used to guide treatment in children with asthma, which is highlighted in the current GINA guidelines. Lung function is a complex trait and reflects a number of structural and functional changes to the airways due to chronic inflammation. It does not correlate with symptom occurrence or severity well, especially in children, as certain patients with poor lung function may not exhibit severe symptoms and vice versa, certain patients with normal lung function may experience symptom aggravation [48,49]. Moreover, children with mild-to-moderate asthma using the controller treatment exhibit a slower decline in lung function in comparison with deterioration of the symptom control [50], which is probably why the model predicts these traits poorly, given the fact that most of the patients in our study had milder disease forms.

Compared to MEF50 and FEV1, FENO performed better in the classification tasks. This suggests that FENO at the baseline can be used as a predictor of steroid responsiveness even more consistently than other parameters, e.g., lung function [20,51]. FENO is a good biomarker of the Th2-related allergic inflammatory response, as interleukin-13 promotes nitric oxide (NO) synthase activity and NO production [52]. Moreover, the latest GINA guidelines [16] suggest that treatment guided by FENO in children and young adults is associated with a significant reduction in exacerbation rates and that it may be a good complementary approach compatible with control-based asthma management. Additionally, since the FENO-based response was able to distinguish true responders quite well, it may be useful in identifying patients with ineffective or suboptimal treatments, those that require treatment adjustment [16] and those with poor adherence to treatment [53].

When comparing MEF50 and FEV1 to each other, MEF50-based outcomes were predicted by FEV1 at the baseline to a lesser extent than FEV1-based outcomes by MEF50 at the baseline. Additionally, MEF50-based outcomes were also predicted by hsCRP (Table 6), which is a marker of subtly elevated systemic inflammation in asthma [40]. The evidence shows that increased hsCRP is associated with more severe asthma outcomes [41]. This, in addition to the fact that the model predicting MEF50-related response performed better in almost all parameters (except specificity) compared to the FEV1-related response (see Table 5) and the fact that oversampling further improved the model’s power in predicting true responders and non-responders (see Figure 2) for MEF50, highlights the importance of the distal airways in children with asthma [42]. The peripheral airways are the predominant site of airway inflammation [43] and may very well be a predominant site of airflow obstruction in asthmatic children, involved in the pathophysiology and resistance to treatment with ICS [44]. Moreover, distal airway impairment may be present despite rare and mild asthma symptoms and normal FEV1 in pediatric patients. The current GINA recommendations are guided by FEV1 (or PEF), but our findings suggest that MEF50 (and other distal airways lung function parameters) should be included as a variable in the diagnostic and assessment processes and guidelines, following more extensive research.

When comparing FENO to LOAC, a lower predictive quality was achieved. This suggests that the model generates a significant proportion of false responders and non-responders for lung function- and FENO-based outcomes, which further supports the control-guided asthma management approach as a preferred option in guiding asthma treatment in children. Additional results by means of the Receiver Operating Characteristic (ROC) curves [54] are presented in Supplementary Figure S2. This highlights the importance of an expert assessment in asthma management from the start, as well as the impact of asthma heterogeneity and its phenotypes for treatment success [55].

The fact that LOAC-based outcomes were predicted predominantly by asthma severity at the baseline suggests that the model recognizes that children with milder disease forms respond better to anti-inflammatory treatment. Additionally, it seems that the model is capable of identifying severe patients quite accurately, but the shortfall is that it does not inform about the potential mechanisms underlying treatment failure. It also seems that prominent markers of atopy (total IgE) are highly predictive of treatment success. The vast majority of childhood asthma patients have allergic asthma, and a number of studies have shown that it is sensitive to treatment with ICS. More specifically, T-helper 2 lymphocyte and T2-high endotypes respond best to ICS [56]. Moreover, since both asthma control and severity encompass the patient’s (caregiver’s) self-estimation on symptom frequency and severity, these findings emphasize the importance of the patient’s involvement in the management plan, an essential part of the “shared-care approach” in asthma management is associated with improved outcomes [16].

Although FENO had a substantially lower accuracy and MCC than LOAC, the treatment outcomes according to the FENO changes were capable of identifying true responders quite well, indicating that the FENO-guided treatment may be a complementary tool in guiding asthma management in children. The permutation importance revealed that predicting FENO is more complex in comparison to LOAC (Table 6). This may be due to the fact that FENO reflects the level and type of airway inflammation that drives the chronicity of the disease. Elevated IgE and sensitization to inhaled allergens are common markers of T2-high inflammation [57] which is known to respond better to anti-inflammatory treatment.

Additionally, a stratification of the patients by age showed that the models exhibited the greatest predictive power in younger children (2–5 years old for LOAC- and MEF50- and 6–11 years old for FENO- and FEV1-based outcomes). The models seemed to perform poorly in older children and adolescents. Age is known to be an important factor in asthma physiology, both age of onset, as well as the duration of the disease. The later age of onset is usually associated with poorer outcomes [58], and the natural course of the disease progressing into adolescence and adulthood (as opposed to remission in puberty) is also associated with the symptoms worsening (although this effect is gender-conditioned) [59,60]. However, having only 10 patients in the 18+ age group might represent a limitation to these findings.

As for the permutation importance, none of the targets involved the available treatment variables, meaning that the models did not use treatment variables in creating decisions on the treatment outcomes. Even though the treatment follows the guidelines, these are not definite nor objective sensu stricto. The guidelines actually provide general choice recommendations, and the physician is left to choose between several treatment options. Although this may represent a potential bias in identifying true responders vs. non-responders, it actually reflects the model’s power of prediction and favors the current symptom of control-guided asthma management approach.

Our results are in concordance with those of Ross et al. [11], who also identified asthma control as the strongest predictive variable for LOAC. These authors only focused on one type of ICS (budesonide) and chromones (nedocromil), while our study encompassed all the commonly used classes of anti-inflammatory controller medication (ICS, LABA and LTRA). Ross et al. [11] only evaluated the response to treatment according to symptom control, while our study involved lung function- and FENO-based treatment outcomes. Although the homogeneity of the population studied (a real-life situation with most of the children having allergic asthma and milder disease forms) could have been an advantage in identifying certain phenotypes and genetic traits associated with the treatment outcomes; this was a disadvantage in identifying clear pathophysiological mechanisms involved. Moreover, the sample size in our study might have been small (*N* = 365 vs. *N* = 1019 in Ross et al. [11]), possibly further hindering a more detailed endotype characterization. Ross et al. identified serum eosinophils as one of the most predictive variables for asthma control, while we identified IgE, supporting our previous findings that children with T2-high allergic asthma respond best to an anti-inflammatory treatment [61]. Finally, even though GINA guidelines suggest a treatment response review every 3–6 months, the assessment period in this study have been too short to reflect the biologically significant and measurable effects, especially on complex traits such as lung function changes in response to treatment.

## 5. Conclusions

Our goal was to evaluate the prediction of treatment outcomes in childhood asthma after six months of medication use. Our results show that asthma control (LOAC) was well-predicted, while the prediction quality of lung function-based treatment outcomes (FEV1 and MEF50) was rather low. These results are in concordance with the GINA control-based management approach, while the lung function may not be the tool of choice to be used in guiding the treatment in children with asthma21. The prediction model for the FENO-based treatment response performed better in almost all aspects compared to lung function-related outcomes, which suggests that treatment success guided by changes in FENO might be a complementary tool in childhood asthma management. Our results also suggest that the current guidelines in asthma management and the current expertise in a clinical assessment (assessment of severity and disease control) are satisfactory in most cases but, also, that our models were quite accurate in identifying patients with ineffective and suboptimal treatment success, which is hardly the case in the current clinical practice. Additionally, our findings emphasize that the distal airway impairment in childhood asthma might have greater diagnostic and assessment value over FEV1.

Although machine learning has shown how the treatment outcome prediction can be driven, it has revealed certain issues that need to be addressed in future studies:

- With respect to asthma chronicity, the assessment period of 6 months may not be enough for valid predictions. Longitudinal and prospective studies are essential;
- Additional studies involving larger numbers of patients with even more clinically relevant parameters are required to increase the success of the treatment outcome prediction and, further, the characterization of specific disease phenotypes and endotypes.

Although machine learning has shown how treatment outcome prediction can be driven, it has revealed certain issues that need to be addressed in future studies:

- With respect to asthma chronicity, the assessment period of 6 months may not be enough for valid predictions. Longitudinal and prospective studies are essential;
- Additional studies involving larger numbers of patients with even more clinically relevant parameters are required to increase the success of the treatment outcome prediction and the further characterization of specific disease phenotypes and endotypes;
- Although we tried to encompass both the objectively measured (lung function and FENO) and subjective target variables (asthma control), a consensus in the choice of the primary study endpoints is required.

Recently, much focus has been given to the implementation of precision medicine in asthma, and experts emphasize that the time for action is now. The use of big data and machine learning in predicting treatment success, such as the one in this study, might enable treatment optimization and the development of new therapies for each defined endotype.

## Figures and Tables

**Table 1:** The variables used in this study, described in more detail in the supplementary file.

Variable Group	Description
demographics	gender, age
subjective clinical data	at baseline (t0)-personal and family medical history-atopy status, allergic rhinitis (AR), atopic dermatitis (AD), food allergy and other comorbidities
objective clinical data	at baseline (t0) and after 6 months (t0 + 6)-symptom control, frequency and severity of exacerbations in the period since the last visit, lung function (FVC, FEV1, MEF50), airway inflammation (FENO) measurement and medication use; at baseline (t0)- skin prick (SPT) and total and specific IgE to inhaled allergens, blood eosinophils and neutrophils, anthropometric measures (height, weight, body mass index) and for certain patients with suggestive history for comorbidities -ENT examination, pH probing with impedance for diagnostics of laryngopharyngeal reflux and gastroesophageal reflux disease, polysomnography for diagnostics of obstructive sleep apnea syndrome, SPT and specific IgE to food and insect venom allergens for diagnostics of food/insect venom allergy
genetic data	genotypes for rs37973 (GLCCI1), rs9910408 (TBX21), rs242941 (CRHR1), rs1876828 (CRHR1), rs1042713 (ADRB2) and rs17576 (MMP9) (see Table S1, Figure S1 and Table S2a,b in the Supplement)

AR: allergic rhinitis, AD: atopic dermatitis, FVC: forced vital capacity, FEV1- forced expiratory volume in one second, MEF50- maximal expiratory flow at 50% of the vital flow capacity, FENO- Fractional Exhaled nitric oxide, SPT: skin prick test, IgE: immunoglobulin E, ENT: ear/nose/throat, GLCCI1: glucocorticoid-induced 1, TBX21: t-box 21, CRHR1: corticotropin releasing hormone receptor 1, ADRB2: beta-2 adrenergic receptor and MMP9: matrix metalloproteinase-9. A full variable list is given in the supplementary file (see Table S3).

**Table 2:** Patient stratification according to their response to treatment (target variables). Response to treatment is defined into more detail in the supplementary file. Ppb: parts per billion.

Class	FEV_1_	MEF_50_	FENO	Asthma Control
**Responders**	Increase ≥ 10% predicted	Increase ≥ 15% predicted	Decrease < 20% for values > 35 (50) ppb or < 10 ppb for values < 35 (50) ppb	Improvement in asthma control
**Non-Responders**	Change < 10% predicted	Change < 15% predicted	Decrease ≤ 20% FENO ≤ 20% for values over 35 (50) ppb or ± 10 ppb for values < 35 (50) ppb or increase >20% for values > 35 (50) ppb or > 10 ppb for values < 35 (50) ppb	No changes in partial asthma control or deterioration in asthma control

FEV1—forced expiratory volume in one second, MEF50—maximal expiratory flow at 50% of the vital flow capacity, FENO—Fractional Exhaled nitric oxide.

**Table 3:** Distribution of the responders and non-responders per measured outcome after 6 months of treatment. The 13 missing FENO response values were imputed. t0 + 6 is the timepoint for 6 months after the start of the treatment.

Treatment Outcome (t0 + 6)	Responders (1)	Non-Responders (0)
LOAC (t0 + 6)	230	135
FENO (t0 + 6)	248	104
FEV1 (t0 + 6)	129	236
MEF50 (t0 + 6)	126	239

FEV1—forced expiratory volume in one second, MEF50—maximal expiratory flow at 50% of the vital flow capacity, FENO—Fractional Exhaled nitric oxide, LOAC—level of asthma control.

**Table 4:** The experimental matrix consists of training two different classifiers × three sampling methods × four targets, meaning there are 24 options (2 × 3× 4), each resampled 100× train–test splitting (giving a total of 600 models trained per target).

1. Classification Algorithm	2. Sampling Methods	3. Targets After Six Months of Treatment
(a) AdaBoost	(a) No sampling (base)	(a) MEF50 (t0 + 6)
(b) Random Forest	(b) Under sampling (cluster centroids)	(b) FEV1 (t0 + 6)
	(c) Oversampling	(c) FENO (t0 + 6)
		(d) LOAC (t0 + 6)

FEV1—forced expiratory volume in one second, MEF50- maximal expiratory flow at 50% of the vital flow capacity, FENO—Fractional Exhaled nitric oxide, LOAC—level of asthma control.

**Table 5:** Average classification results for the treatment targets FEV1, FENO, MEF50 and LOAC. The results are reported for the best performing model (classifier and sampling method) and are calculated by the mean of the accuracy, specificity, sensitivity and the MCC.

	FEV1	FENO	MEF50	LOAC
Accuracy	0.6503	0.7005	0.6753	0.9698
Specificity	0.8986	0.8531	0.8817	0.9661
Sensitivity	0.7854	0.9560	0.7855	0.9781
MCC	0.2190	0.2146	0.2608	0.9366

MCC is the Matthews correlation coefficient. FEV1—forced expiratory volume in one second, MEF50—maximal expiratory flow at 50% of the vital flow capacity, FENO—Fractional Exhaled nitric oxide, LOAC—level of asthma control.

**Table 6:** Top important variables for each of the targets. The variables were aggregated by the median value of the permutation importance per target (600 runs each). The permutation importance is divided by the respective Matthews correlation coefficient (MCC) value from Table 5 and calculated as the % of weight respective to the MCC, i.e., contribution to MCC. For each target, only several variables returned an aggregated median above 1%. hsCRP: high-sensitivity C-reactive protein and t0: baseline.

Variable	LOAC	FENO	FEV1	MEF50
Seasonal allergens (SPT)		1.1%		
Asthma severity (t0)	47.0%			
hsCRP				1.2%
IgE total		1.5%	3.2%	
FENO (t0)		12.8%		
FEV1 (t0)			14.8%	1.8%
MEF50 (t0)			8.2%	30.3%

**Table 7:** Analysis of model results stratified by age. Each MCC value is the median of 200 models. The patients are grouped in age groups (2–5, 6–11, 12–17 and 18+). The number of patients per age group is given in the column “No. Patients”. Superscripts b and w are used to describe the best and worst results per response, respectively.

Response	Age Group	MCC (All Models)	No. Patients
LOAC	2–5 y/o	^b^ 1	53
	6–11 y/o	0.96	178
	12–17 y/o	0.89	124
	>18 y/o	^w^ 0.61	10
FENO	2–5 y/o	^w^ 0	53
	6–11 y/o	^b^ 0.14	178
	12–17 y/o	0.1	124
	>18 y/o	^w^ 0	10
FEV1	2–5 y/o	0.12	53
	6–11 y/o	^b^ 0.27	178
	12–17 y/o	0.08	124
	>18 y/o	^w^ 0	10
MEF50	2–5 y/o	^b^ 0.29	53
	6–11 y/o	0.18	178
	12–17 y/o	0.25	124
	>18 y/o	^w^ 0	10

No. Patients—number of patients, y/o—years old.

**Table 8:** Comparison of the ensemble models to logistic regression. The median MCC values across all the models grouped by the target variables are compared.

Response	Sampling	Logistic Regression	AdaBoost	Random Forest
FENO	Base	0.07	0.21	0.07
	CC	−0.02	0.07	0.10
	OS	0.05	0.17	0.13
FEV1	Base	0.00	0.22	0.14
	CC	0.03	0.14	0.15
	OS	0.03	0.18	0.19
LOAC	Base	0.19	0.94	0.90
	CC	0.04	0.89	0.90
	OS	0.17	0.93	0.90
MEF50	Base	−0.01	0.23	0.19
	CC	−0.02	0.14	0.12
	OS	0.03	0.24	0.26

CC—cluster centroids, OS—oversampling (both explained in Section 2.3).

## Data Availability

There is no consent to data sharing.

## Supplementary Materials

The following are available online at https://www.mdpi.com/article/10.3390/children8050376/s1, Figure S1: Genotype frequencies, Figure S2: ROC curves and confusion matrices, Table S1: Primers (forward and reverse) and probes (allele 1 and allele 2) design for rs17576 genotyping assay, Table S2: (a) HWE consistency for genotype frequencies for rs37973, rs9910408, rs242941, rs1876828, rs1042713 and rs17576, (b) Observed and expected genotype frequencies for rs37973, rs9910408, rs242941, rs1876828, rs1042713 and rs17576, Table S3: All predictive variables (X), Table S4: Confusion matrix.
