# Supplementary material for: Predicting Treatment Outcomes Using Explainable Machine Learning in Children with Asthma
Source: Children (Basel). 2021 May 10;8(5):376. doi: 10.3390/children8050376 (PMC8151683; doi:10.3390/children8050376)
Supplement: Supplementary file 1 [file children-08-00376-s001.zip › children-1196519-supplementary.pdf]

Predicting treatment outcomes using explainable machine learning in children with asthma

Mario Lovrić [1,&], Ivana Banić [2,&], Emanuel Lacić [1], Kristina Pavlović [1], Roman Kern [1,3,*] and Mirjana Turkalj [2,4,5,*]

1 Know-Center, Infeldgasse 13, 8010 Graz, Austria; mlovric@know-center.at (M.L.), elacic@know-center.at (E.L.), kpavlovic@know-center.at (K.P.)

2 Srebrnjak Children's Hospital, Srebrnjak 100, 10000 Zagreb, Croatia; ibanic@bolnica-srebrnjak.hr (I.B.)

3 Graz University of Technology, Inffeldgasse 16C, 8010 Graz, Austria

4 Faculty of Medicine, J.J. Strossmayer University of Osijek, Josipa Huttlera 4, 31000 Osijek, Croatia

5 Catholic University of Croatia, Ilica 242, 10000 Zagreb, Croata

& These authors contributed equally.

* Correspondence: mturkalj@bolnica-srebrnjak.hr , Tel.:+385 1 6391 191 (HR, M.T.); rkern@know-center.at Tel.: +43 316 873 - 30860 (AT, R.K.)

Supporting information

Table of Contents

Observational study description....3

Inclusion criteria....3

Exclusion criteria....4

Follow up....4

Assessments....4

Potential bias....5

Diagnostic data description....5

Assessment and diagnostic tests/procedures....5

Genetic analysis....6

Genotyping results....7

Definition of treatment outcomes....10

Definition of response according to changes in lung function....10

Definition of response to treatment according to airway inflammation....10

Definition of response to treatment according to asthma control (LOAC)....11

Classification metrics....11

Additional figures....13

Supplement literature....14

Observational study description

The controlled and observational (non-interventional) 6-months study with prospective cohort design and longitudinal data collection was performed in paediatric patients with physician (paediatric pulmonologist or allergy specialist) established diagnosis of asthma, at the Srebrnjak Children`s Hospital in Zagreb, Croatia. The routine standardized follow-up assessments of all patients included- First patient (or study subject), First Visit (FPFV): 1 month after the beginning of the study- Last Patient (or study subject), First Visit: 1-12 months- Last Patient (or study subject), Last Visit: 6-12 months- End of Study (Including follow up and data analysis); 24 months. The study size was estimated at 400, with a dropout rate after the 24-month follow-up period being ca. 10% (N=365).

Inclusion criteria

The study included patients with signed informed consent, i.e. those who have agreed to participate in a clinical study and met the inclusion criteria assessed by a paediatric pulmonologist or allergy specialist. The children were enrolled in the study after their legal guardians have read, agreed to, and signed the informed consent form. All children older than 12 as well as adolescents were enrolled in the study after signing the inform consent, as well. The subjects were assessed for inclusion and exclusion criteria on first study visit after signing the informed consent. Patients involved in the study used their regular treatment (according to GINA guidelines)[1], i.e., conduct treatment and all other non-therapeutic measures as if they were not involved in the study (real life study). Any deterioration or emergency interventions were evaluated and recorded as additional visits. New therapies were not applied to study patients during the observational period and the patients continued with their prescribed treatment (according to GINA guidelines)[1] as if they were not included in the study. Patients with a clinical diagnosis of asthma (according to ERS/ATS guidelines) for at least one year [1], being on a stable dose of anti-inflammatory treatment for at least one month with partially controlled or uncontrolled asthma according to GINA guidelines[1] were recruited from the SCH outpatient clinic during their regular visit to the clinic (real life study). Additional inclusion criteria were: clinically significant allergy to indoor and/or outdoor allergens, with positive skin prick test (SPT) and/or specific IgE levels (>0.35 kUA/L). SPT was performed to a standard palette of inhaled allergens containing: birch, hazel, grass pollen, mugwort, Ambrosia elatior, *Dermatophagoides pteronyssinus* (Der p), *Dermatophagoides farinae* (Der f), dog, cat, Cladosporium, Alternaria, with negative control (saline) and positive histamine hydrochloride control, 1.0 mg/mL (Allergopharma,

Reinbek, Germany). If indicated, patients underwent SPT to food allergens: eggs, cow's milk, wheat flour, soy, peanut, fish, nuts and sesame, as well as other food allergens (if indicated). The interpretation of SPT results (based on the guidelines of the European Academy of Allergy and Clinical Immunology, EAACI), were made after approximately 15–20 min in relation to positive and negative control. The wheal diameter of at least 3 mm greater than negative control was considered a positive result, according to EAACI recommendations[2]. SPT was not repeated if the respondent had a test finding that was not older than one year since their enrolment in the study.

Exclusion criteria

Exclusion criteria for adolescent patients were: acute respiratory infection, use of systemic corticosteroids, recent asthma-related visit to emergency department (in the past three weeks) and coexistence of other serious chronic illness and smoking cigarettes. Exclusion criteria for preschool and school age children were: known inborn or perinatal pulmonary disease; pulmonary malformation; oxygen therapy after birth with a duration of more than 24 h; ventilator support or mechanical ventilation after birth; diagnosis of cystic fibrosis; primary ciliary dyskinesia; congenital heart disease, chronic lung diseases such as e.g., interstitial lung disease. Moreover, children were excluded from study visits and biomaterial collection in the case of fever of at least 38.5 °C during the last two weeks prior to the first study visit or follow-up.

Follow up

All patients were followed up after 6 months of continuous treatment use. Lung function testing, FENO and asthma control (including C-ACT score) were recorded and assessed at this visit.

Assessments

Lung function (school aged children and younger, if cooperative) by standard spirometry was performed according to the ATS/ERS spirometry standards.[3] We measured forced vital capacity (FVC), forced expiratory volume at the first second (FEV1), FEV1/FVC ratio and mid forced expiratory flow (FEF25-75) and express values as absolute ones and as percentage of the predicted values according to Quanjer. Fraction of exhaled nitric oxide (FENO) was measured using a chemiluminescence analyzer (NIOX or MEDISOFT analyzer) during a single-breath exhalation according to the ERS/ATS recommendations at a flow rate of 50 mL/sec.[4]

Potential bias

Potential bias included overrepresentation of one gender (male) since childhood asthma is more common in boys. During the recruitment procedure, children were included in the study regardless of their gender, if they met the inclusion criteria. Additionally, to avoid under- or over-estimation of the disease diagnosis and state (disease severity and control), the recruitment was performed by experienced clinicians (allergy or pulmonary specialist with 10+ years of experience in the field). Although children of all age groups (4-18) were recruited to the study if they met the inclusion criteria, a diagnosis of asthma was difficult to establish in young children (wheeze, transient wheeze and other differential diagnoses), a potential bias is underrepresentation of younger children (up to 5 years of age).

Diagnostic data description

Assessment and diagnostic tests/procedures

At their first visit patients underwent physical examination, anthropometric measurements (height and weight for calculation of BMI and BMI percentiles), along with a standard battery of diagnostics procedures and measurements to establish a diagnosis of particular asthma phenotype and endotype. These included skin prick tests for common allergens, lung function tests (including airway challenge or bronchodilator tests) and blood sampling for routine laboratory diagnostics (differential blood count, allergy assays- total and allergen-specific IgE). In order to assess the level and type of both systemic and local inflammation, certain inflammatory biomarkers were measured at baseline in all participants, including fractional exhaled nitric oxide (FENO), high-sensitive C-reactive protein (hsCRP) as well as certain inflammatory cell counts, such as eosinophils and neutrophils.[4,5] In order to identify additional conditions that might affect and aggravate the underlying disease (and asthma control), the participants were evaluated for common asthma comorbidities, such as allergic rhinitis, gastroesophageal reflux disease (GERD), laryngopharyngeal reflux (LPR), food allergy, obstructive sleep apnoea syndrome (OSAS) as well as atopic dermatitis and standardized diagnostic procedures.[6,7] Peripheral whole blood samples were collected by venipuncture into EDTA coated vacutainers (for hematology analyses) and into vacutainers with clot activator and gel for serum separation (for biochemistry and certain allergy assays). During this study a total amount of 10 mL peripheral blood samples per participant maximum was collected at baseline visit (recruitment point). The remainder of blood samples (in EDTA

coated vacutainers) and sera left over after diagnostic tests was stored at -20°C for subsequent analyses, including genotyping.

Genetic analysis

All genetic loci analysed in this study were chosen due to their previous association with treatment outcomes in patients with asthma (and childhood asthma, where applicable) in an effort of replicating these associations.

Genomic DNA was extracted from EDTA-containing peripheral whole blood samples, previously stored at -20°C, using QIAamp DNA Blood Mini Kit (Qiagen GmbH, Germany) according to the manufacturer's instructions, by an automated (Qiacube, Qiagen GmbH, Germany) or manual spin protocol. The quality and concentration of each DNA isolate was checked using a NanoDrop™ 2000 spectrophotometer (ThermoFisher Scientific Inc., USA).The genotypes of the SNPs analysed were determined using a 5`-nuclease allelic discrimination assay in a 96-well format and Taqman technology. Primers and probes were purchased from Applied Biosystems (Life Technologies, USA) for SNP genotyping assays rs37973 in *GLCCI1*, rs9910408 in *TBX21* and rs242941 and rs1876828 in *CRHR1*, as well as rs1042713 in *ADRB2*. Allelic discrimination assays were performed in 5 μL reaction volumes, using approximately 5 ng of DNA as a template, 2x TaqMan Fast Advanced Master Mix, and predesigned SNP genotyping assays provided by Applied Biosystems for rs37973, rs9910408, rs242941 and rs1876828. Temperature conditions for qPCR were set at 50°C for 2 minutes and 95°C for 20 seconds, followed by 40 cycles at 95°C for 3 seconds and at 60°C for 30 seconds. For rs17576 SNP (*MMP9*) genotyping a primer and probe set were designed using a free online software qPCR primer & probe design tool and design service (Eurofins Genomics, Germany). The forward and reverse primer as well as probe sequences are presented in Table S1.

Table S1. Primers (forward and reverse) and probes (allele 1 and allele 2) design for rs17576 genotyping assay (*MMP9* Gln279Arg, A/G transition detection). FWD- forward primer, REV- reverse primer, Allele 1 PR- probe for SNP allele 1, Allele 2 PR- probe for SNP allele 2, bp- base pairs (length of sequence in base pairs), reporter- fluorescent dye, quencher- fluorescent dye quencher pair. For probe sequences nucleotides highlighted in red denote the ambiguity position (transition).

Oligonucleotide type	Length (bp)	Sequence	Reporter/ quencher
**MMP9 Gln279Arg FWD primer**	19	TCCCCCTTTCCCACATCCT	
**MMP9 Gln279Arg REV primer**	21	CAGGGTTTCCCATCAGCA TTG	
**MMP9 Gln279Arg Allele 1 PR**	17	CTCTACACCC**A**GGACGG	VIC-BHQ1
**MMP9 Gln279Arg Allele 2 PR**	17	TCTACACCC**G**GGACGG	FAM-BHQ1

Allelic discrimination assays for rs1042713 and rs17576 was performed in 12.5 μL reaction volume, using approximately 5 ng of DNA template (2x Brilliant III Ultra-Fast QPCR Master Mix (Agilent Technologies, USA), 100 mM of primer set (forward and reverse) and 10 mM of probes, with qPCR conditions as follows: 50°C for 2 minutes and 95°C for 10 minutes, followed by 40 cycles at 95°C for 15 seconds and at 60°C for 1 minute.

Genotyping of the amplified PCR products was determined by differences in VIC and FAM fluorescent levels, using the ABI Prism 7500 Fast Real-Time PCR system (system instrument equipped with SDS v2.0.5 software, Applied Biosystems, ThermoFisher Scientific Inc., USA) for rs37973, rs9910408, rs242941 and rs1876828 and using the Agilent AriaMX Real-Time PCR system (system instrument equipped with AriaMx software v1.0, Agilent Technologies, USA) for rs1042713 and rs17576.

Genotyping results

Genotype distribution (frequency) for each genetic polymorphism is shown in Figure s1. All participants were successfully genotyped for rs37973, rs9910408, rs242941. For rs1042713 and rs17576 genotype data was missing for 1 and 19 participants, respectively, due to insufficient DNA extract material or degraded DNA samples in subsequent/ repeated analysis. Genotyping was performed at the Srebrnjak Children`s Hospital in Zagreb, Croatia in 2 batches on 2 different platforms.

Figure S1. Genotype frequency (%) for respective genetic polymorphisms: rs37973, rs9910408, rs242941, rs1876828, rs1042713 and rs17576. For rs37973, rs9910408, rs242941 and rs1876828 N=365, for rs1042713 N=364 and for rs17576 N=346.

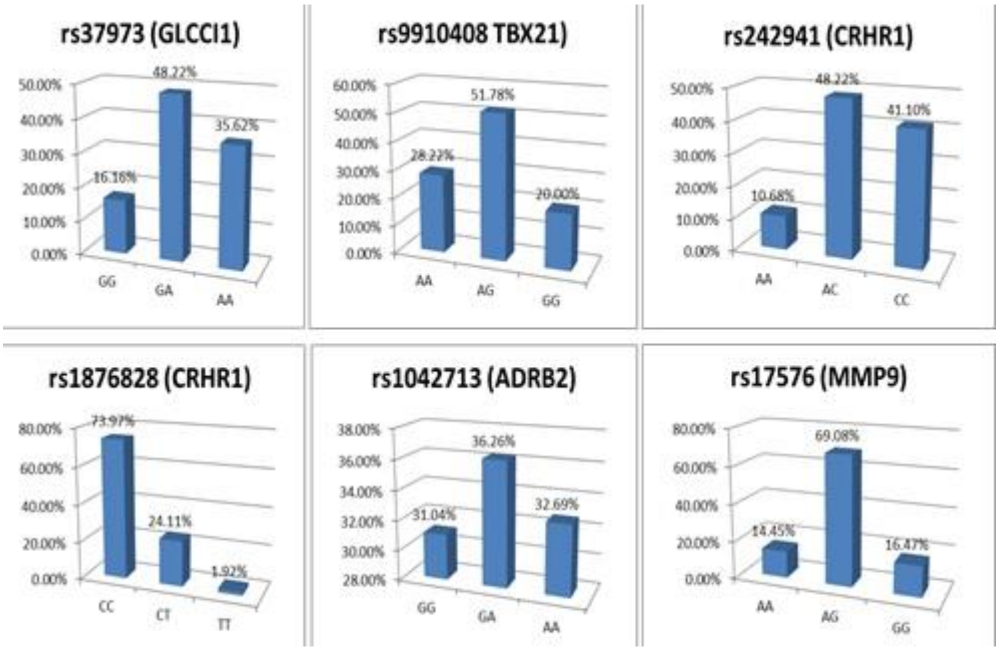

Consistency with the Hardy- Weinberg equilibrium (HWE) for each genetic polymorphism, along with global and population-specific minor allele frequency (MAF) are presented in Table S2a and S2b.

Table S2a. HWE consistency for genotype frequencies for rs37973, rs9910408, rs242941, rs1876828, rs1042713 and rs17576. χ^2– chi-squared value, $p < 0.05$ consistent with HWE. Global and population specific (Central European) MAF according to NCBI dbSNP (https://www.ncbi.nlm.nih.gov/SNP/). HWE- Hardy-Weinberg equilibrium, MAF- minor allele frequency, CEU- Central European, reference population. Calculated using Michael H. Court's (2005-2008) online calculator[8].

Genotype	rs37973	rs9910408	rs242941	rs1876828	rs1042713	rs17576
Variant allele frequency	0.40	0.46	0.35	0.14	0.51	0.51
χ^2	0.002	0.664	1.433	0.003	27.433	50.508
p (1 degree of freedom)	0.965	0.415	0.231	0.956	0.000	0.000
Global MAF	0.396	0.384	0.323	0.086	0.476	0.456
CEU MAF	0.442	0.456	0.282	0.240	0.358	0.381

Table S2b. Observed and expected genotype frequencies for rs37973, rs9910408, rs242941, rs1876828, rs1042713 and rs17576. Calculated using Michael H. Court's (2005-2008) online calculator[8].

Genotype	rs37973		rs9910408		rs242941		rs1876828		rs1042713		rs17576	
	Observed	Expected	Observed	Expected	Observed	Expected	Observed	Expected	Observed	Expected	Observed	Expected
Homozygote reference	130	130.2	103	106.9	150	155.2	270	270.1	113	88.0	50	83.0
Heterozygote	176	175.6	189	181.3	176	165.6	88	87.7	132	182.0	239	172.9
Homozygote variant	59	59.2	73	76.9	39	44.2	7	7.1	119	94.0	57	90.0

Definition of treatment outcomes

According to their response to specific classes of treatment (ICS alone, LTRA alone, ICS+LABA and/or LTRA), patients were divided into "responders" and non-responders in accordance with the American Thoracic Society (ATS) and European Respiratory Society (ERS) task forces` interpretation of changes in lung function (FEV1 and MEF50)- the MCID for lung function adjusted for children (% of predicted lung function) as well as data from other studies evaluating treatment response in asthma- by taking into account changes in the level of disease control (according to GINA), as well as changes in the level of airway inflammation, i.e. FENO values[1,3,4].

Definition of response according to changes in lung function

Treatment non-responders according to changes in FEV1 were defined as those having a decrease in FEV1 predicted (for children of certain age, sex and posture- height, weight) by 10% or more between clinical visits/follow-ups (≤10%), whereas responders were defined as those having an increase in FEV1 predicted by 10% or more (≥10%) between clinical assessments/follow-ups.

Treatment non-responders according to changes in MEF50 were defined as those with a decrease in MEF50 predicted (for children of certain age, sex and posture) by 15% or more (≤15%), whereas responders were defined as those having an increase in MEF50 predicted by 15% or more (≥15%) between clinical visits/follow-ups.[9,10]

Definition of response to treatment according to airway inflammation

According to ATS recommendations, cut points rather than reference values were used when interpreting FENO levels[4]: low FENO (25 ppb in adults; 20 ppb in children), high FENO (50 ppb in adults, 35 ppb in children), intermediate FENO (between 25 ppb and 50 ppb in adults; 20–35 ppb in children). Treatment non-responders were defined as those having an increase in FENO greater than 20% for values over 35 (50 for patients older than 18 years) ppb or more than 10 ppb for values lower than 35 (50) ppb between clinical visits or a reduction of ≤20% and increase ≤20% for FENO values over 35 (50) ppb and ±10 ppb for values lower than 35 (50) ppb from one visit to the next. Participants having a reduction of at least 20% in FENO for values

over 35 (50) ppb or more than 10 ppb for values lower than 35 (50) ppb were defined as responders.

Definition of response to treatment according to asthma control (LOAC)

The level of asthma control between clinical visits was assessed according to GINA guidelines[1]: taking into account symptom occurrence (including nocturnal symptoms), need for reliever medications (namely SABA), number and severity of asthma exacerbations, lung function and Asthma Control Test (ACT), where applicable. The level of control was defined as either controlled, partly controlled or uncontrolled, whereas treatment non-responders were defined as those having a deterioration in asthma control between visits (controlled to uncontrolled or partly controlled) as well as no changes in partial asthma control and uncontrolled asthma from visit to visit. Participants exhibiting an improvement in asthma control (partly- or uncontrolled to controlled) and those having their asthma controlled between visits were defined as responders.

Classification metrics

A confusion matrix (**Table S3**) is a representation of the output of a classifier that allows to draw conclusions on the model's behaviour, especially if it able to cope with imbalanced data. In this work we assigned responders as a positive (1) class and non-responders to a treatment as negative (0)[11].

Table S3. Description of confusion matrix used for binary classification performance evaluation. Columns represent true values, positive and negative (responders and non-responders), while the rows represent prediction outputs. The optimal output would have only true positives and true negatives. Classification metrics (equations 1 to 5) are calculated from the confusion matrix. Classification algorithms susceptible to class imbalances with often result in too high values for the majority class at the expense of the minority class.

**Predicted class**			
**Responders (1)**	TP (True Positives or True Responders)	FP (False Positives or False Non-Responders)	
**Non-Responders (0)**	FN (False Negatives or False Responders)	TN (True Negatives or True Non-Responders)	
	Responders (1)	Non-Responders (0)	True class

Accuracy is a metric commonly used in classification. Accuracy (eq. 1) is the proportion of patients being correctly predicted (classified) over the total amount of patients.

$$Accuracy = \frac{TP + TN}{TP + TN + FP + FN} \qquad (1)$$

Accuracy is considered ineffective in cases with an underrepresented class.[12,13] The reason is that falsely estimated patients (*FN*, *FP*) are considered only in the denominator and will have an insignificant role if *TP* or *TN* are large. **Sensitivity** (eq. 2) is the number of true positives that are correctly classified. One can consider it a measure of completeness (i.e. number of patients correctly identified as positive out of total true positive patients).

$$Sensitivity = \frac{TP}{TP + FN} \qquad (2)$$

Specificity represent the true negative rate (eq. 3) We use specificity to better assess the predictive capability of non-responders and sensitivity to assess the predictive capability of responders.

$$Specificity = \frac{TN}{TN + FP} \qquad (3)$$

In both sensitivity and specificity, the influence of *FP* and *FN* are more pronounced than in accuracy. The **Matthews correlation coefficient** (eq. 4) is a correlation coefficient for binary

variables[13,14], which in contrast to accuracy also takes falsely estimated patients into consideration. For perfect classification (FP = FN = 0) the value of MCC is 1, meaning perfect positive correlation. If the classifier has misclassified (TP = TN = 0), it will yield is -1, representing perfect negative correlation. As such, the value of MCC falls between -1 and 1, with 0 indicating a random result. We use MCC to better assess the overall model quality if one of the classes in underrepresented.

$$MCC = \frac{TP*TN-FP*FN}{\sqrt{(TP+FP)*(TP+FN)*(TN+FP)*(TN+FN)}} \qquad (4)$$

Additional figures

Figure S2. Left) ROC curves for the best model per target. This is done in by plotting the true positive against the false positive rate (as defined in Table 5). The desired effect in this regard is to get the highest possible area under the curve (AUC). Actually, getting a straight line would mean an area of 0.5 and be equal to a classifier that makes random predictions (similar to coin tossing). In our case, we see that the highest prediction quality is achieved for the LOAC treatment outcome. Right) Confusion matrices for the best model per target with average values for TP, TN, FN, FP. The confusion matrices are obtained for 100 runs for each best model described in the manuscript.

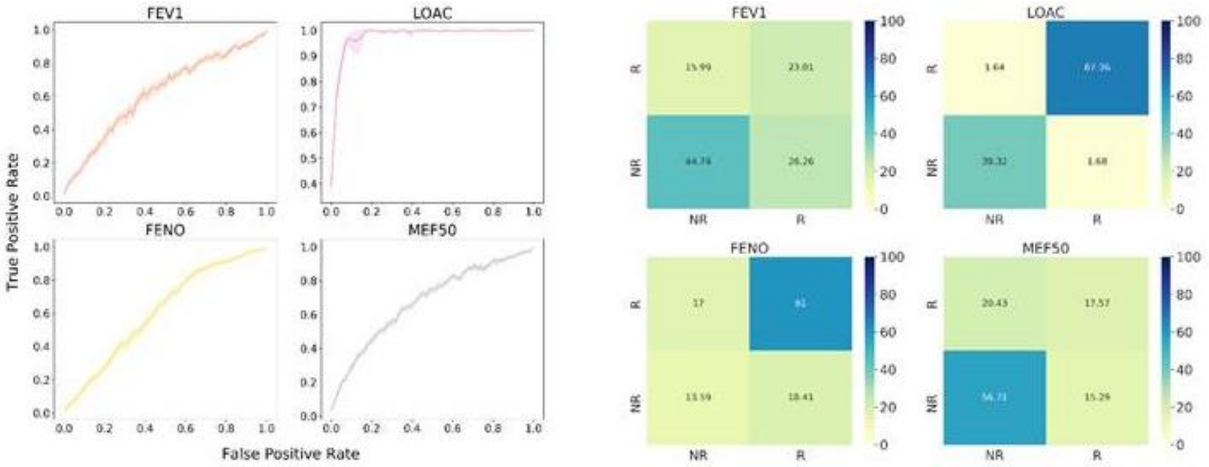

Supplement literature

1. Global Initiative for Asthma. Global Strategy for Asthma Management and Prevention, 2020. [Internet]. 2020. Available from: http://www.ginasthma.org/

2. Heinzerling L, Mari A, Bergmann KC, Bresciani M, Burbach G, Darsow U, et al. The skin prick test – European standards. Clin Transl Allergy. 2013;3:3.

3. Reddel HK, Taylor DR, Bateman ED, Boulet L-P, Boushey HA, Busse WW, et al. An Official American Thoracic Society/European Respiratory Society Statement: Asthma Control and Exacerbations. Am J Respir Crit Care Med. 2009;180:59–99.

4. Dweik RA, Boggs PB, Erzurum SC, Irvin CG, Leigh MW, Lundberg JO, et al. An Official ATS Clinical Practice Guideline: Interpretation of Exhaled Nitric Oxide Levels (F e $_{NO}$) for Clinical Applications. Am J Respir Crit Care Med. 2011;184:602–15.

5. Navratil M, Plavec D, Dodig S, Jelcic Z, Nogalo B, Erceg D, et al. Markers of systemic and lung inflammation in childhood asthma. J Asthma. 2009;46:822–8.

6. Streets CG, DeMeester TR. Ambulatory 24-hour esophageal pH monitoring: why, when, and what to do. J Clin Gastroenterol. 2003;37:14–22.

7. Hrvatsko pedijatrijsko društvo. R, Sabol Z, Turkalj M, Nenadić N, Marušić I, Kučić D, et al. Paediatria Croatica : časopis Hrvatskoga pedijatrijskog društva i Hrvatskog društva za školsku i sveučilišnu medicinu za pedijatriju, adolescentnu medicinu i granične struke. Vol. 57, Paediatria Croatica. Klinika za dječje bolesti; 2013.

8. Court MH (2012) Court's (2005–2008) online calculator. Accessed: http://emerald.tufts.edu/~mcourt01/Documents/Court%20lab%20-%20HW%20calculator.xls on 3rd August 2018.

9. Telenga ED, van der Berge M, ten Hacken NHT, Riemersma RA, van der Molen T, Postma DS (2013) Small airways in asthma: their independent contribution to the severity of hyperresponsiveness. Eur Respir J 41: 752-754.

10. Boskabady M, Rezaeitalab F, Rahimi N, Dehnavi D (2008) Improvement in symptoms and pulmonary function of asthmatic patients due to their treatment according to the Global Strategy for Asthma Management (GINA). BMC Pulm Med 8: 26.

11. Chicco D, Rovelli C. Computational prediction of diagnosis and feature selection on mesothelioma patient health records. PLoS One. 2019;14.

12. Chicco D. Ten quick tips for machine learning in computational biology. Vol. 10, BioData Mining. BioMed Central Ltd.; 2017.

13. Lučić B, Batista J, Bojović V, Lovrić M, Sović Kržić A, Bešlo D, et al. Estimation of Random Accuracy and its Use in Validation of Predictive Quality of Classification Models within Predictive Challenges. Croat Chem Acta. 2019;92.

14. Boughorbel S, Jarray F, El-Anbari M. Optimal classifier for imbalanced data using Matthews Correlation Coefficient metric. PLoS One. 2017;12.
